# Elevated whole muscle phosphatidylcholine: phosphatidylethanolamine ratio coincides with reduced SERCA activity in murine overloaded plantaris muscles

**DOI:** 10.1186/s12944-018-0687-7

**Published:** 2018-03-13

**Authors:** Val A. Fajardo, John S. Mikhaeil, Cameron F. Leveille, A. Russell Tupling, Paul J. LeBlanc

**Affiliations:** 1 Department of Health Sciences, Brock University, St. Catharines, ON L2S 3A1 Canada; 2 Centre for Bone and Muscle Health, Faculty of Applied Health Sciences, Brock University, St. Catharines, ON Canada; 3 Department of Kinesiology, University of Waterloo, Waterloo, ON Canada

**Keywords:** Phospholipid, Skeletal muscle, Overload

## Abstract

**Background:**

An increase in phosphatidylcholine:phosphatidylethanolamine (PC:PE) and a decrease in fatty acyl chain length, monounsaturated:polyunsaturated (MUFA:PUFA) fatty acyl ratio reduces SERCA activity in liposomes and in mouse models of obesity and muscular dystrophy. We have previously shown that maximal SERCA activity is significantly reduced in mechanically overloaded (OVL) plantaris, however, whether changes in PC:PE ratio or fatty acyl composition may contribute to the alterations in maximal SERCA activity remain unknown. Here, we tested the hypotheses that in OVL plantaris 1) PC:PE ratio would negatively correlate with maximal SERCA activity and 2) PC fatty acyl chain length (ACL) and/or MUFA:PUFA ratio would positively correlate with maximal SERCA activity.

**Methods:**

To overload plantaris in mice, we transected the soleus and gastrocnemius tendons from one leg, while the contralateral leg underwent a sham surgery. After two weeks, plantaris muscles were extracted, homogenized and processed for SERCA activity and lipid analyses. Specifically, we performed HPTLC densitometry to examine changes in PC, PE, and the ratio of PC:PE. We also performed gas chromatography to assess any potential changes to fatty acyl composition.

**Results:**

SERCA activity was significantly reduced in OVL plantaris compared with sham. Coinciding with this, we found a significant increase in PC but not PE in OVL plantaris. In turn, there was an increase in PC:PE but did not reach significance (*p* = 0.09). However, we found a significant negative correlation between PC:PE and maximal SERCA activity. Fatty acyl composition of PE remained similar between OLV and sham and PC demonstrated higher percent mole fraction of 17:1, 18:1, and ACL compared to sham. In addition, PC ACL, % MUFA, % PUFA, or MUFA:PUFA did not significantly correlate with maximal SERCA activity.

**Conclusions:**

Our results indicate that the phospholipid headgroup PC:PE negatively correlated and could potentially contribute to reductions in SERCA activity seen in functionally overloaded plantaris. In contrast, fatty acyl chain (ACL, % MUFA, % PUFA, MUFA:PUFA) did not correlate with maximal SERCA activity. Future studies will determine whether altering PC:PE with genetic and dietary interventions can influence SERCA activity and ultimately change the physiological outcome in response to muscle overloading.

## Introduction

The sarco(endo)plasmic reticulum Ca^2+^-ATPase (SERCA) pumps are 110 kDa integral membrane proteins that catalyze the re-uptake of Ca^2+^ from the cytosol into the sarcoplasmic reticulum (SR), thereby eliciting muscle relaxation and maintaining low intracellular calcium ([Ca^2+^]_i_) [1, 2]. Over the last few years, there has been a renewed interest in the study of membranes in regulating SERCA activity. Novel developments in X-ray crystallography have revealed that membrane phospholipids are indeed key components of SERCA pump function [3]. Two phospholipids in particular are phosphatidylethanolamine (PE) and phosphatidylcholine (PC), which together represent the major phospholipids in skeletal muscle [4]. It is well-established that increasing the PC:PE ratio reduces SERCA function [5, 6], and may have implications in obesity and insulin resistance [7, 8], and muscular dystrophy [9].

We [10] and others [11] have shown that maximal SERCA activity is significantly reduced in functionally overloaded rodent plantaris muscles. Many factors may contribute to this reduction in SERCA activity including: an increased proportion of slow-oxidative fibers [12], which have lower SERCA pump density [13]; and an increase in sarcolipin protein, which is known to negatively regulate the Ca^2+^ pump [14]. However, to our knowledge, no study to date has examined whether changes in PC:PE ratio could also influence SERCA function in this model. Building from our previous work [10], we mechanically overloaded plantaris in mice by tenotomizing soleus and gastrocnemius muscles in order to examine the hypothesis that reductions in SERCA activity in overloaded plantaris would correlate with increases in PC:PE ratio. In addition, previous research has demonstrated that longer chain length and greater level of monounsaturates compared to polyunsaturates leads to increased SERCA activity in synthetic lipid environments [5, 15]. As such, we also examined the fatty acid composition of PC and PE through gas chromatography to test whether any changes in PC and/or PE acyl chain length or level of unsaturation would be correlated with SERCA activity. Thus, we hypothesized that in mechanically overloaded plantaris 1) PC:PE ratio would negatively correlate with maximal SERCA activity and 2) PC fatty acyl average chain length and higher monounsaturates compared to polyunsaturates would positively correlate with maximal SERCA activity.

## Methods

### Mice

The mice used in this study were part of a previously published study examining changes in mitochondrial membrane lipid analyses in response to muscle overloading and unloading stimuli [16]. Specifically, six adult (4–6 month) male C57BL/6 mice (29.1 ± 1.2 g) were used and housed in an environmentally controlled room with a standard 12:12-h light-dark cycle and allowed access to food and water ad libitum. All animal procedures were reviewed and approved by the Brock University Animal Care and Utilization Committee and are consistent with the guidelines established by the Canadian Council on Animal Care.

### Plantaris mechanical overload

To overload plantaris, mice were first anaesthetized with 2% isoflurane in a precision vaporizer. Next, soleus and gastrocnemius tendons were transected in one leg while the other contralateral leg served as a sham control [14, 16]. Mice were then left for two weeks in individually housed cages to adapt as we have demonstrated previously that this time frame results in significant plantaris hypertrophy [16]. Subsequently, mice were anaesthetized in an induction chamber using isoflurane (5%), transitioned to a nose cone (5% isoflurane) and then placed on a surgical bed for muscles to be surgically removed. Once removed, plantaris muscles were then homogenized in homogenizing buffer (250 mM sucrose, 5 mM HEPES, 0.2 mM PMSF, 0.2% [*w*/*v*] NaN_3_) in a 10:1 ratio (*v*/*w*) and stored at − 80 °C until further analysis. Once the muscles were removed, the mice were sacrificed via exsanguination while anesthetized with isoflurane.

### SERCA activity

SERCA activity was assessed in plantaris homogenates over Ca^2+^ concentrations ranging from *p*Ca 7.1 to 5.3 in the presence of the Ca^2+^ ionophore A23187 (Sigma C7522) using a Ca^2+^-dependent, enzyme-linked spectrophotometric plate reader assay that has been described previously [17]. Maximal SERCA activity was taken from the raw data and SERCA activity-*p*Ca curves were generated with GraphPad Prism™ by non-linear regression curve fitting using an equation for a general cooperative model for substrate activation.

### Absolute quantification of PC and PE lipids

**Fig. 1:**
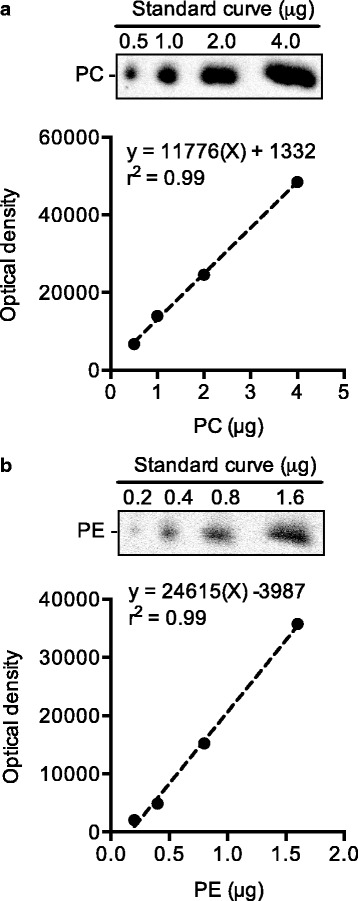
Representative images of PC (**a**) and PE (**b**) standard curves that were loaded onto each HPTLC plate

Total lipids from plantaris homogenates (1.25 mg) were extracted as previously described [18] and were subsequently spotted onto high-performance thin layer chromatography plates (HPTLC; 5633–5, EMD Chemicals, Darmstadt, Germany) and individual phospholipids were separated using a chloroform:methanol:acetic acid:water (100:75:7:4) solvent system [4]. A standard curve (0.5, 1.0, 2.0, 4.0 μg) of purified PC (P3556, Sigma Aldrich, MO, USA), and a standard curve (0.2, 0.4, 0.8, 1.6 μg) of purified PE (P7943, Sigma Aldrich) were also loaded onto each HPTLC plate. After allowing the solvent to run up each plate for 45 min, the plates were then charred at 180 °C with a 10% (*w*/*v*) copper (II) sulfate in 8% phosphoric acid solution for 15 min [19]. Images of the HPTLC plates were captured using a CCD camera on a Fluorchem 5500 imaging station (Alpha Innotech, CA, USA) under reflective white light. Densitometry analyses were then performed using imageJ (National Institutes of Health, MA, USA) and the standard curve of PC and PE (Fig. 1) were used to calculate the absolute PC and PE concentrations (per 1.25 mg of muscle) in plantaris.

### PC and PE fatty acid composition

The fatty acyl composition of PC and PE were measured as previously described [20]. Total lipids were extracted from plantaris homogenates (1.25 mg), then spotted onto a separate HPTLC plate, and allowed to run in the same solvent and for the same time as discussed in the previous section. Next, the separated phospholipids on the HPTLC plates were identified by spraying a dichlorofluoroscein (DCF) solution (methanol:water [1:1], and 2′,7’-DCF filtered and washed with petroleum ether) onto the plate and setting it into a chamber containing 25% ammonium hydroxide for 5 min. The HPTLC plate was viewed under ultraviolet light and PC and PE bands were marked and scraped into individual 15 ml kimex culture tubes and allowed to methylate in 2 ml of 6% H_2_SO_4_ (*w*/*v* in methanol) overnight at 50 °C. Fatty acyl methyl esters were extracted with petroleum ether, dried down, reconstituted into dichloromethane (20 μl), and injected (2 μl) into a gas chromatograph (Trace GC Ultra, Thermo Electron, Milan, Italy) fitted with a split/splitless injector, a fast flame ionization detector, and Triplus AS autosampler (Trace GC Ultra, Thermo Electron) as previously described [20]. PC and PE fatty acyl methyl esters were separated on an UFM RTX-WAX analytical column (Thermo Electron) using helium as a carrier gas. Fatty acids were identified by comparison of retention times with those of a known standard (Supelco 37 component FAME mix, Supelco, PA, USA), and absolute amounts (nmol) were calculated with the aid of the internal standard, tridecanoic acid (13:0), which was added to the samples immediately prior to the methylation process. The percent mol fraction of the individual fatty acid species was calculated using the sum of the absolute amounts as the denominator.

### Statistics

All values presented here are means ± standard error (SE). All comparisons were made using a paired t-test and a Pearson’s correlation was performed to test the association between maximal SERCA activity and PC:PE ratio, PC ACL, PC % MUFA, PC % PUFA, and PC MUFA:PUFA. Statistical significance was set to *p* ≤ 0.05.

## Results

### Muscle morphometrics

As previously reported, the overloaded plantaris muscles from the mice used in this study exhibited significant muscle hypertrophy with a 75% increase in muscle mass and a 1.8-fold increase in plantaris:body weight ratio [16]. In a recently published study, we showed that the increase in plantaris muscle mass due to the overload surgery was attributed to an increase in total myofibre number and cross-sectional area [10].

### Maximal SERCA activity is reduced in the overloaded plantaris

**Fig. 2:**
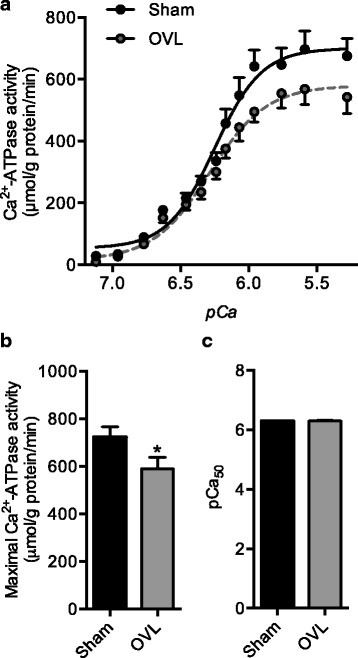
SERCA activity in sham and overloaded (OVL) plantaris muscles. (**a**) Representative SERCA activity-*p*Ca curves. (**b**) Maximal SERCA activity is reduced in the overloaded plantaris. (**c**) SERCA’s apparent affinity for calcium is unaltered in the overloaded plantaris compared with sham. *Significantly different from sham, *p* ≤ 0.05; (*n* = 6 per group). *p*Ca_50_ is the negative logarithm of [Ca^2+^] required to elicit half maximal SERCA activity

Consistent with our recent observations in this model [14], we observed a significant 20% reduction in maximal SERCA activity in overloaded (OVL) plantaris compared with sham (Fig. 2a and b). Furthermore, *p*Ca_50_ was unaltered between sham and OVL plantaris muscles (Fig. 2c).

### PC but not PE increased significantly in overloaded plantaris and PC:PE ratio negatively correlated with maximal SERCA activity

**Fig. 3:**
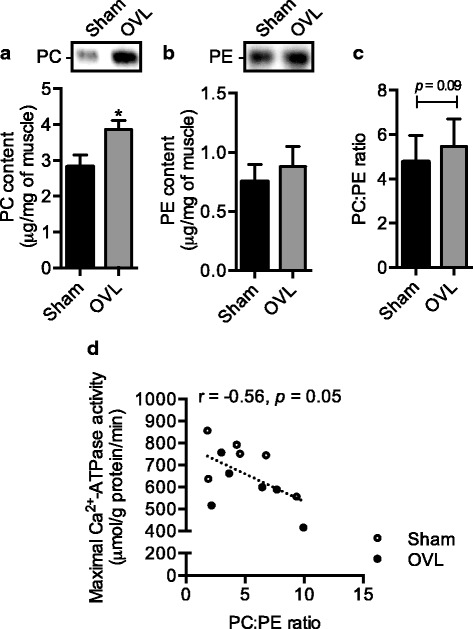
Elevated PC (**a**) but not PE (**b**) leads to a trending increase in PC:PE ratio (**c**) in the overloaded (OVL) plantaris compared with sham, and the PC:PE ratio is negatively correlated with maximal SERCA activity (**d**). For (A)-(C), *significantly different from sham, *p* ≤ 0.05; (*n* = 6 per group)

When examining the changes in PC and PE individually, we found that there was a significant 37% increase in PC (Fig. 3a), whereas there was no significant change in PE levels (Fig. 3b). In turn, and in agreement with our hypothesis, we detected a 15% increase in the PC:PE ratio in OVL plantaris compared with sham, however this did not reach significance (*p* = 0.09, Fig. 3c). However, plotting PC:PE ratio against maximal SERCA activity rates revealed a significant negative correlation (Fig. 3d).

### PC fatty acyl chain length but not the level of unsaturation significantly increased in overloaded plantaris and none were correlated to maximal SERCA activity

**Fig. 4:**
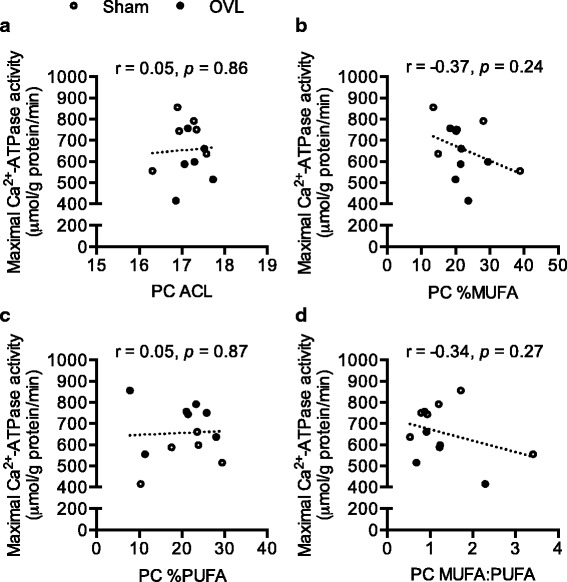
PC ACL (**a**), % MUFA (**b**), % PUFA (**c**), and MUFA:PUFA (**d**) correlational analyses with maximal SERCA activity (*n* = 6 per group)

For both PC and PE, 16:0 and 18:0 were the major fatty acid species representing 35–45% of all fatty acids; but there were no changes in the percent mol fractions of these fatty acids in either PC or PE between sham and OVL plantaris (Table 1). We did detect a significant increase in PC 17:1, PC 18:1, and PC ACL in overloaded plantaris compared with sham (2%, 3%, and 1.5%, respectively) (Table 1). PC ACL, % MUFA, % PUFA, and MUFA:PUFA did not significantly correlate with maximal SERCA activity (Fig. 4).

**Table 1:** Phosphatidylcholine (PC) and phosphatidylethanolamine (PE) fatty acid composition in sham and overloaded plantaris muscles (*n* = 6 per group)

	PC		PE	
	Sham	OVL	Sham	OVL
14:0	3.5 ± 0.88	2.7 ± 0.4	3.7 ± 0.9	2.9 ± 0.8
16:0	33.5 ± 3.8	31.2 ± 2.5	19.1 ± 4.6	22.1 ± 3.5
17:0	3.9 ± 0.8	3.5 ± 0.6	5.7 ± 1.1	4.6 ± 0.8
18:0	11.9 ± 2.9	11.8 ± 1.0	18.9 ± 4.2	24.6 ± 4.3
14:1	4.3 ± 1.7	2.1 ± 0.8	2.0 ± 0.3	2.0 ± 0.4
16:1	5.0 ± 0.8	4.9 ± 0.4	4.0 ± 0.8	3.8 ± 1.1
17:1	2.8 ± 0.6	4.7 ± 0.9*	6.2 ± 1.3	4.6 ± 1.5
18:1	5.0 ± 0.8	7.9 ± 0.8*	7.1 ± 1.0	8.4 ± 1.6
18:2*n*6	5.0 ± 0.9	6.3 ± 0.8	2.2 ± 0.4	2.6 ± 0.5
20:2*n*6	1.7 ± 0.7	2.3 ± 1.2	3.6 ± 1.8	1.85 ± 0.7
20:3n3	4.3 ± 1.0	5.0 ± 0.9	1.9 ± 0.7	2.1 ± 0.6
20:5*n*3	0.3 ± 0.2	0.3 ± 0.1	0.2 ± 0.1	0.6 ± 0.3
22:6n3	4.4 ± 1.3	3.5 ± 0.7	5.4 ± 1.7	4.3 ± 1.5
SFA	57.7 ± 4.5	56.6 ± 2.9	56.2 ± 5.7	60.8 ± 5.7
MUFA	22.6 ± 3.9	22.4 ± 1.6	26.6 ± 3.9	22.3 ± 3.6
PUFA	19.6 ± 3.3	21.0 ± 2.7	17.1 ± 2.9	16.9 ± 3.0
*n*3	9.7 ± 2.3	9.2 ± 1.6	8.1 ± 2.4	7.5 ± 2.2
*n*6	8.3 ± 1.2	10.5 ± 1.7	7.6 ± 1.9	8.2 ± 1.7
MUFA:PUFA	1.4 ± 0.4	1.2 ± 0.2	1.7 ± 0.2	1.4 ± 0.3
ACL	17.1 ± 0.2	17.3 ± 0.1*	17.3 ± 0.2	17.4 ± 0.2

All values are means ± SE. *Significantly different from sham *p* ≤ 0.05. SFA, saturated fatty acids; MUFA, monounsaturated fats; PUFA, polyunsaturated fats; ACL, average chain length, which is calculated as Σ(*m*_i_ x *c*_i_), where *m*_i_ is the molar concentration and *c*_i_ is the number of carbon atoms

The units listed are in percent mole fraction

## Discussion

In the present study, we sought to determine whether changes in PC:PE ratio could potentially contribute to the reductions in maximal SERCA activity we previously observed in overloaded plantaris [14]. Here, we found that a significant increase in PC but not PE led to a non-significant increase in PC:PE ratio, and that the PC:PE ratio negatively correlated with maximal SERCA activity. In all, our data are consistent with previous studies indicating a potential role for PC:PE ratio in the regulation of SERCA activity [7–9, 21]. Although the exact mechanism by which an increase in PC:PE ratio decreases SERCA activity is unknown, increasing %PE in synthetic liposomes steadily increases maximal SERCA activity [5]. Furthermore, Gustavsson et al. [5] found that increasing %PE in liposomes also increased SERCA’s affinity for Ca^2+^; which is thought to be mediated through greater covalent interactions between PE and SERCA [6]. Notably, we did not detect any significant differences in SERCA’s apparent affinity for Ca^2+^ between sham and OVL plantaris; however, our assay does not directly assess SERCA’s capacity to bind Ca^2+^. Future research should examine the influence of overload-mediated changes on SERCA’s Ca^2+^ binding capacity.

We also examined the fatty acyl composition of PC and PE in response to muscle overloading. OVL plantaris resulted in increased PC 17:1, 18:1, and ACL compared to sham. Despite changes in two of the four MUFAs detected, this did not result in significant changes in % MUFA. In addition, PC ACL, % MUFA, % PUFA, and MUFA:PUFA did not significantly correlate with maximal SERCA activity. These findings are in contrast to synthetic liposome models, where SERCA activity is greater when reconstituted in PC membranes with longer ACL and higher levels of monounsaturation, particularly 18:1 [5]. Gustavsson et al. [5] have suggested that a monounsaturated bilayer of 20–22 carbons long results in maximal SERCA’s V_max_ and Ca^2+^ affinity. The discrepant results may be explained by 1) the small yet statistically significant increase in PC 17:1, 18:1, and ACL did not alter SERCA activity potentially due to the larger influence of PC:PE; and 2) the fact that synthetic liposomes do not reflect the complexity of skeletal muscle SR, which is made up of 60–75% PC, 10–25% PE, 9–10% PI, and 2–3% PS [22–25]. As such, our results suggest that from a lipid perspective in naturally occurring membranes, it may be the ratio between PC and PE headgroups rather than any changes in fatty acyl configuration that may contribute to the reduction in SERCA activity seen with muscle overload stimuli.

A limitation to our study is that we examined the PC:PE ratio in whole muscle homogenates rather than purified SR membranes. Although HPTLC is highly sensitive and is capable of measuring lipids from purified SR membranes, in our hands, this would require a large amount of tissue (~ 100 mg), where often we would pool muscles from multiple rats [22]. Thus, we were limited in our approach to quantify SR PC and PE in this study given the relatively small muscle sizes from mice (sham, 15.4 ± 1.3 mg vs. OVL, 26.9 ± 0.7 mg [16]) . Although we acknowledge the fact that whole muscle lipid analyses may mask potential differences that occur specifically at the subcellular level [4, 26, 27], our study inherently operates under the assumption that changes in whole muscle PC:PE ratio is reflective of the changes that occur in the SR. Indeed, in the basal state, isolated SR PC (60–75%) and PE (10–25%) make up the majority of phospholipids [22–25], which is similar to what is observed in whole muscle [4]. Here, our densitometry analyses in sham plantaris indicate that PC and PE account for 55% and 23% of the total lipids, respectively. Furthermore, because we were able to detect a significant negative correlation between whole muscle PC:PE ratio and SERCA activity, this could potentially negate the need for isolating SR membranes for PC:PE analyses. However, future studies should determine whether whole muscle PC:PE ratio is indeed reflective of SR PC:PE ratio. In addition, future studies should assess the temporal changes between PC:PE ratio and SERCA activity to determine whether it is truly changes in PC:PE ratio that mediates changes in SERCA function or whether changes in SERCA activity precede changes in PC:PE ration and thus could be mediating the changes in membrane lipid composition.

## Conclusions

In conclusion, the results reported here regarding PC:PE ratio during plantaris overloading stimuli is in support of previous studies demonstrating its role in regulating SERCA in skeletal muscle. Future studies aimed at altering PC:PE ratio through genetic and dietary interventions should be performed to further examine its role in regulating SERCA function and its potential physiological impact.

## Abbreviations

FAS: Fatty acid synthase
MUFA: Monounsaturated fatty acids
PC: Phosphatidylcholine
PE: Phosphatidylethanolamine
PUFA: Polyunsaturated fatty acids
SERCA: Sarco (endo) plasmic reticulum Ca^2+^-ATPase
SFA: Saturated fatty acids
SR: Sarcoplasmic reticulum
UI: Unsaturation index
